# Foxp3 Silencing with Antisense Oligonucleotide Improves Immunogenicity of an Adjuvanted Recombinant Vaccine against *Sporothrix schenckii*

**DOI:** 10.3390/ijms22073470

**Published:** 2021-03-27

**Authors:** Alexander Batista-Duharte, Luis Sendra, Maria José Herrero, Deivys Leandro Portuondo, Damiana Téllez-Martínez, Gladys Olivera, Manuel Fernández-Delgado, Beatriz Javega, Guadalupe Herrera, Alicia Martínez, Paulo Inacio Costa, Iracilda Zeppone Carlos, Salvador Francisco Aliño

**Affiliations:** 1 Department of Clinical Analysis, School of Pharmaceutical Sciences, São Paulo State University (UNESP), Araraquara 14800-903, São Paulo, Brazil; deivysleandro@gmail.com (D.L.P.); damianatellezm@gmail.com (D.T.-M.); costapi.unesp@gmail.com (P.I.C.); iracilda.zeppone@unesp.br (I.Z.C.); 2 Pharmacology Department, Faculty of Medicine, Universitat de Valencia, 46010 Valencia, Spain; Maria.Jose.Herrero@uv.es (M.J.H.); gherreramartin@gmail.com (G.H.); salvador.f.alino@uv.es (S.F.A.); 3 Pharmacogenetics Unit, Instituto de Investigación Sanitaria La Fe, 46026 Valencia, Spain; gladysmjiv@hotmail.com; 4 Service of Hematology and Hemotherapy, Hospital General Universitario de Castellón, 12004 Castelló de la Plana, Spain; mfdezdm@gmail.com; 5 Cytometry Unit, Faculty of Medicine, Universitat de Valencia, 46010 Valencia, Spain; beatriz.javega@uv.es; 6 Cytomics Unit, Centro de Investigación Príncipe Felipe, 46012 Valencia, Spain; amartinez@cipf.es; 7 Unit of Clinical Pharmacology, Medicine Clinical Area, Hospital Universitario y Politécnico La Fe, 46026 Valencia, Spain

**Keywords:** antisensense oligonucleotide, Foxp3, regulatory T cells, vaccine immunogenicity, *Sporothrix schenckii*

## Abstract

Background: In recent years, there has been great interest in developing molecular adjuvants based on antisense oligonucleotides (ASOs) targeting immunosuppressor pathways with inhibitory effects on regulatory T cells (Tregs) to improve immunogenicity and vaccine efficacy. We aim to evaluate the immunostimulating effect of 2′OMe phosphorothioated Foxp3-targeted ASO in an antifungal adjuvanted recombinant vaccine. Methods: The uptake kinetics of Foxp3 ASO, its cytotoxicity and its ability to deplete Tregs were evaluated in murine splenocytes in vitro. Groups of mice were vaccinated with recombinant enolase (Eno) of *Sporothix schenckii* in Montanide Gel 01 adjuvant alone or in combination with either 1 µg or 8 µg of Foxp3 ASO. The titers of antigen-specific antibody in serum samples from vaccinated mice (male C57BL/6) were determined by ELISA (enzyme-linked immunosorbent assay). Cultured splenocytes from each group were activated in vitro with Eno and the levels of IFN-γ and IL-12 were also measured by ELISA. The results showed that the anti-Eno antibody titer was significantly higher upon addition of 8 µM Foxp3 ASO in the vaccine formulation compared to the standard vaccine without ASO. In vitro and in vivo experiments suggest that Foxp3 ASO enhances specific immune responses by means of Treg depletion during vaccination. Conclusion: Foxp3 ASO significantly enhances immune responses against co-delivered adjuvanted recombinant Eno vaccine and it has the potential to improve vaccine immunogenicity.

## 1. Introduction

Regulatory T cells (Tregs) are a subset of CD4+ T-cells that play a suppressive role in the immune system. Tregs control the immune response to self and foreign antigens, helping to prevent over-inflammation and autoimmune disease [1,2,3]. On the other hand, a great deal of evidence shows that Tregs are often involved in the failure of anti-infectious defense [4,5,6,7,8] and effective vaccination [9,10,11,12,13].

In recent years, there has been great interest in developing molecular adjuvants with inhibitory effects on Tregs, aiming to improve the immunogenicity and vaccine efficacy [14,15] against deleterious foreign antigens. One of the newer strategies for depletion/inhibition of Tregs in vaccines has been the use of antisense oligonucleotides (ASOs) targeting important immunosuppressor pathways or immune checkpoints [16]. ASOs are small-sized (around 20 nucleotides) single-stranded oligonucleotides designed to bind specifically to the RNA or DNA target, based on their sequence homology, and bring about gene silencing [17]. Several ASOs have already been approved by the United States Food and Drug Administration (FDA) and many others to treat cardiovascular, metabolic, endocrine, neurological, neuromuscular, inflammatory, and infectious diseases [18] are under study in clinical trials.

Foxp3 (forkhead box P3), also known as the scurfin protein, is a member of the forkhead transcription factor family that is mainly expressed in Tregs. Foxp3 acts as a transcription activator for several genes, such as CD25, Cytotoxic T-Lymphocyte Antigen 4 (CTLA-4), glucocorticoid-induced TNF receptor family gene (GITR), and folate receptor 4. The regulation of Foxp3 expression in Tregs occurs through the concerted action of transcription factors, epigenetic control mechanisms, and post-translational modifications that modulate Foxp3 function [19]. Vaccination of mice with dendritic cells transfected with Foxp3 mRNA promoted selective depletion of Foxp3+ Tregs and stimulated specific cytotoxic T lymphocytes (CTL) with enhanced vaccine-induced protective immunity [20]. An improvement of vaccine immunogenicity and Foxp3 targeting efficacy has been reported using other strategies, including a chimeric Foxp3-Fc(IgG) fusion construct/protein to stimulate the immune responses against Tregs [21], as well as synthetic peptides with the ability to inhibit Foxp3 function [22,23,24].

In a previous study by our group, therapeutic vaccination was combined with either Foxp3 or CTLA4 gene silencing to enhance the antitumor response following B16 tumor cell transplantation. Either 2’-O-methyl phosphorotioate-modified oligonucleotides (2’-OMe-PS-ASOs) or polypurine reverse Hoogsteen hairpins (PPRHs) were used for Foxp3 or CTLA4 gene silencing. Combining the therapeutic vaccine with Foxp3 ASO achieved a greater survival rate (50%) than with CTLA4 ASO (20%), associated with Treg depletion. In that study, both ASOs were injected intraperitoneally, and the pharmacological effects were observed only at the higher doses [25]. Thus, the high cost and the potential risk of off-target effects and toxicity limited this strategy [26].

In this study, we evaluated whether a low dose of ASO Foxp3 as part of the vaccine formulation could improve vaccine immunogenicity. We studied the kinetics of Foxp3 ASO access into CD4+ T cells. The functional effects of the ASO on Treg depletion, cytotoxicity, and its ability to enhance the immunogenicity of an antifungal adjuvanted vaccine against sporotrichosis [27] were also evaluated.

## 2. Results

### 2.1. Primary Sequence of the Foxp3 Gene in FASTA Format and Target Region for Foxp3 Silencing

The target sequence of the ASO used for silencing the Foxp3 gene was located in intron 1 of the last update of NCBI (Supplement Figure S1). This location allowed us to estimate that its possible site of action could be at the pre-RNA level, still containing the non-coding sequences. Therefore, the probable silencing mechanism could occur through the action of an RNAse-H, which cleaves the heterodimer, or also by a steric hindrance mechanism and splicing inhibition [16].

### 2.2. Oligonucleotide (ON) Uptake Kinetics in Murine Splenocytes

**Figure 1:**
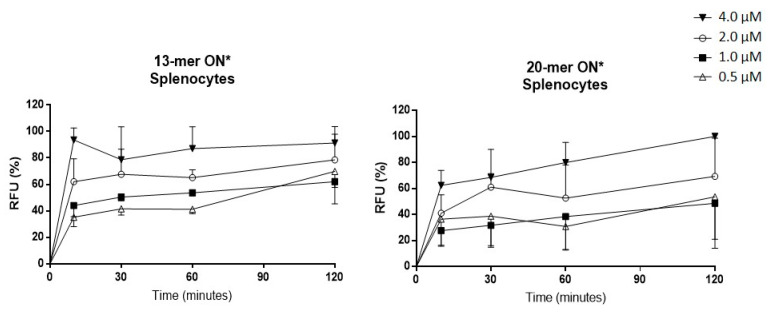
Labeled oligonucleotide (ON) absorption kinetics in splenocytes. Labeled ONs with sizes of 13 or 20 mer were cultured at different times and concentrations in the presence of C57BL6 mouse splenocytes. The fluorescent ONs of either 13 or 20 mer, labeled with Cy5 or FITC, were incubated independently at concentrations of 0.5, 1, 2, and 4 µM at 37 °C at 0, 10, 30, 60, and 120 min. At each time, the splenocytes were analyzed by means of flow cytometry to measure the ON uptake and the % of ONs was calculated with respect to maximal relative fluorescence unit (RFU) values achieved. ON* refers to fluorescent oligonucleotide.

Before the in vivo studies, the optimal conditions for the ON uptake were determined with a size-range study that encompassed those normally used for gene silencing, such as the anti-Foxp3 ASO used in this study. The ability of two fluorescent ONs with different sizes (13-mer and 20-mer) to enter splenocytes was evaluated. Fluorescence was determined by means of flow cytometry at different times between 0 and 120 min, employing different concentrations (0.5, 1.0, 2.0, and 4.0 µM). As shown in Figure 1, after 10 min of incubation, most of the ONs could be detected in the cells. From that time on, although the entry of ONs into the cells continued to increase at concentrations of 0.5, 1, and 2 µM, at concentrations of 4 and 10 µM from 10 min of incubation no greater absorption of ONs was observed. Regarding the sizes of the ONs, no differences were observed between the 13 and 20 mer ONs, and splenocytes and lymphocytes showed a similar pattern of uptake. This result coincides with other studies carried out in our laboratory using human blood in which similar absorption patterns were observed (results not-shown); thus, both mouse splenocytes and human blood models can be used for studies of ON uptake kinetics.

**Figure 2:**
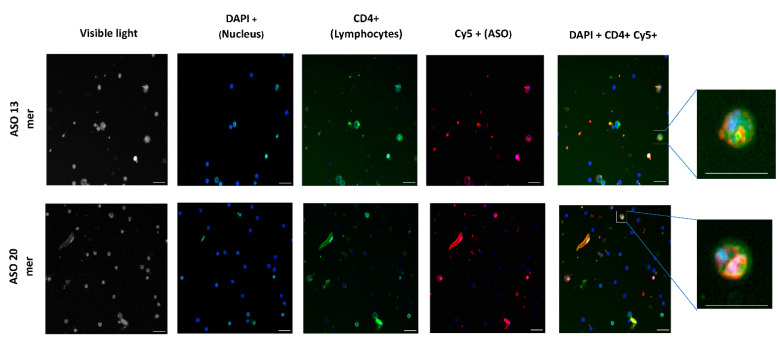
Location of fluorescent ONs in splenic lymphocytes. Splenocytes were treated with Cy5-labeled ONs and incubated for 1 h at 4 µM. Then, the cells were marked with anti-mouse CD4 FITC, fixed with 4% formaldehyde in phosphate buffered saline (PBS) solution and analyzed using an InCell analyzer 2200 system to observe the uptake of ONs in CD4+ lymphocytes. Scale bar represents 10 µm.

To determine the possible cellular location of the labeled ONs, an imaging study was carried out using InCell equipment. As shown in Figure 2, the presence of lymphocytes with labeled ONs in their cellular structure was confirmed, although it was not possible to precisely define their cellular location.

### 2.3. Biological Activity of the ASO Foxp3

**Figure 3:**
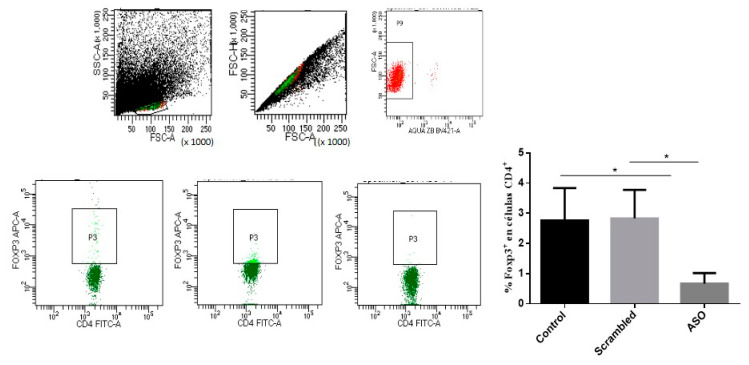
Biological activity of the anti-Foxp3 antisense oligonucleotide (ASO). Effect of anti-Foxp3 ASO in mouse splenocytes on CD4 + Foxp3 + cell populations. The upper panel represents the strategy of gates and the confirmation of the viability of the cells studied. The lower panel shows representative images of the reduction of CD4+ Foxp3+ population (as %) in the cells treated with anti Foxp3 ASO. A one-way analysis of variance (ANOVA) with Tukey’s post-hoc test was used. The confidence interval was established at 95% for all tests. The level of significance and *p*-values are shown as * (*p* < 0.05).

To confirm the silencing effect of the anti-Foxp3 ASO on this transcription factor, B16 cells from murine melanoma, which constitutively express high levels of Foxp3, were cultured and incubated with anti-Foxp3 or Scrambled ONs. Considering the results of the previous studies, it was determined to use a concentration of 2 µM and incubate it for 1 h. After incubation, a 1:4 dilution with complete RPMI (Roswell Park Memorial Institute) medium was performed and cells were kept in culture for 48 h at 37 °C and 5% CO_2_. Under these conditions, Foxp3 mRNA expression was silenced by about 70%. Moreover, mouse splenocytes were cultured with either anti-Foxp3 or scrambled ASO and the presence of CD4 + CD25 + Foxp3 cells was measured. A significant reduction from ~3% to ~1% of Tregs (*p* < 0.05) was observed with Foxp3 ASO treatment, as evidence of Treg depletion in the culture (Figure 3).

### 2.4. Cellular Viability

**Figure 4:**
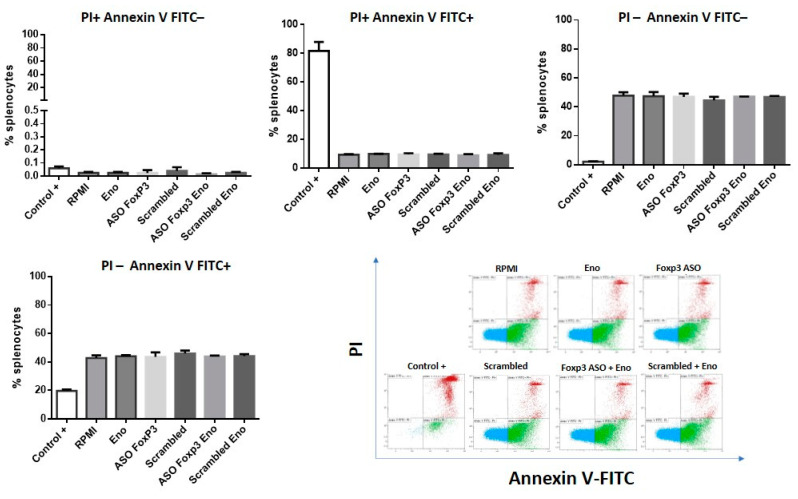
Analysis of variance (ANOVA) with Tukey’s post-hoc test was used. The confidence interval was established at 95% for all tests. There were no significant differences between the experimental groups. A positive heat-killed cell control was used by means of incubation at 56 °C for 30 min.

Next, the cytotoxicity of Eno, anti-Foxp3 ASO, and its scrambled control alone or in combination was evaluated. The concentrations used were the same as those employed in the functional studies and were incubated for 48 h as well. Cell viability was analyzed using a combination of PI/Annexin V-FITC to determine the presence of necrosis (PI+ Annexin V−), late apoptosis (PI+ Annexin V+) and living cells (PI− Annexin V− or PI− Annexin V+). Late apoptosis cells (PI+ Annexin V+) suffer irreversible damage, whereas PI− Annexin V+ cells are in early apoptosis, which can be reversible. As shown in Figure 4, live cells (both PI− Annexin V− and PI− Annexin V+) accounted for more than 90% in all cells treated with Eno, ASO anti-Foxp3, scrambled or in their combinations. This result showed that the direct cytotoxicity of both molecules at the concentrations employed is low, and that they can be used in in vitro studies.

### 2.5. Immunogenicity Study

With these initial results, an experimental study to evaluate the effect of the administration of anti-Foxp3 ASO on the immunogenicity of an experimental vaccine against *Sporothrix schenckii* previously developed in our laboratory [27,28] was carried out. We used the same experimental design previously described but including the anti-Foxp3 ASO within the formulation to determine its effect on the specific immune response. Two doses of anti-Foxp3 ASO, 1 or 8 µg per mouse and dose, were used to evaluate a possible dose-response effect.

#### 2.5.1. Anti-Eno Antibodies

**Figure 5:**
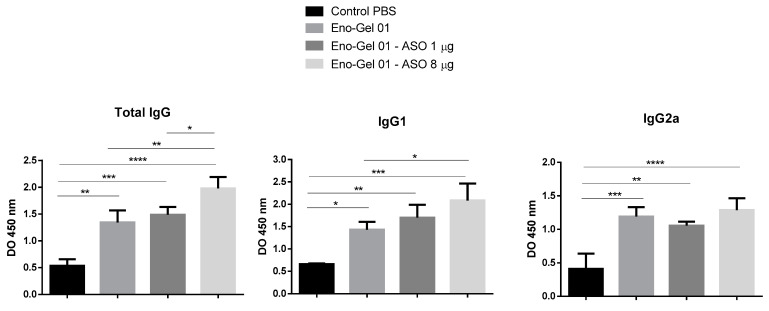
Specific antienolase antibodies. Mice (C57BL/6) were immunized on days 0 and 14. Specific antibodies to *S. schenckii* enolase in serum were evaluated on day 21 by means of ELISA. A one-way analysis of variance (ANOVA) with Tukey’s post hoc test was used. The confidence interval was established at 95% for all tests. The level of significance and *p*-values are shown as * (*p* < 0.05); ** (*p* < 0.01); *** (*p* < 0.001); **** (*p* < 0.0001).

To evaluate the antibody response, the serum was extracted from the animals’ blood samples and the titers of total IgG, IgG1, and specific IgG2a were quantified. As can be seen in Figure 5, the groups immunized with the vaccine using adjuvant and ASO at 8 µg presented higher titers of specific IgG and IgG1 antibodies. In the case of IgG1, a dose-response trend could be observed. For IgG2a, significant differences between the groups with and without ASO were not observed.

This result shows that anti Foxp3 ASO mediated greater stimulation of the immune response, suggesting that the inhibition of the suppressive effect of Tregs could have helped to achieve a greater immunogenicity of enolase. Other authors have obtained similar results using other ASOs targeting Tregs [29,30,31,32]. Further studies will be necessary to confirm these findings.

#### 2.5.2. CD4+ CD25+ Foxp3 T Cells

**Figure 6:**
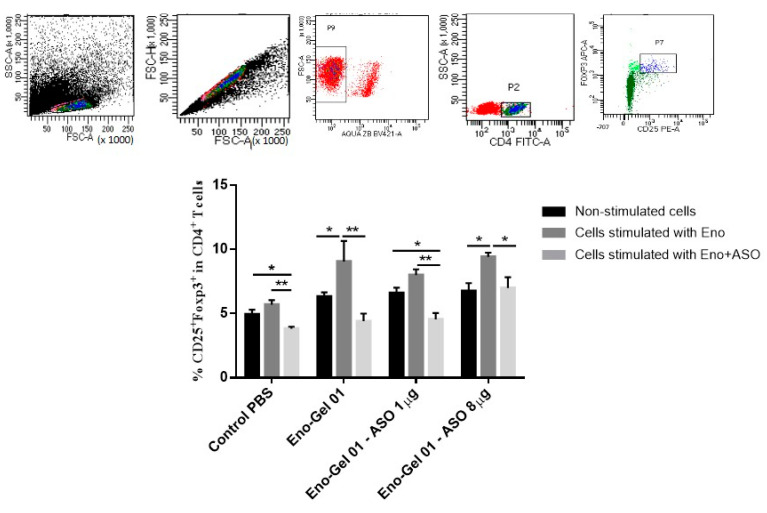
CD4+ CD25+ Foxp3 T cells in mouse spleens. Splenocytes were extracted on day 21 after immunization, then they were stimulated for 48 with Eno of *S. schenckii* or with Eno + ASO anti-Foxp3. Treg % was evaluated by means of flow cytometry. Top panel shows the gates strategy used in the study. A one-way analysis of variance (ANOVA) with Tukey’s post hoc test was used. The confidence interval was established at 95% for all tests. The level of significance and *p*-values are shown as * (*p* < 0.05); ** (*p* < 0.01).

To evaluate the effect of the different immunization regimens on Tregs, splenocytes were cultured for 48 h in the presence of Eno or Eno+ anti-Foxp3 ASO. As shown in Figure 6, the vaccinated groups exhibited a higher presence of Tregs after stimulation in vitro than non-vaccinated groups. However, there were no differences between the group vaccinated with Eno-Gel 01 and the groups treated with Eno and ASOs. Similarly, cells cultured with Eno + ASO showed Treg reductions compared to cells that were only stimulated with Eno, although this reduction was lower in the group immunized with the highest dose of ASO anti-Foxp3.

#### 2.5.3. IFN-γ and IL-12 Production

**Figure 7:**
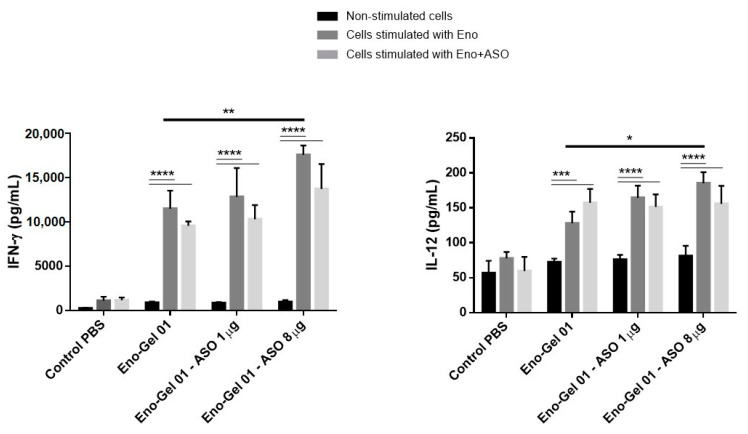
IFN-γ and IL-12 cytokines in the spleen. The spleen cells were extracted on day 21, then incubated for 48 h with Eno of *S. schenckii* or with Eno+ ASO anti-Foxp3. The cytokines IFN-γ and IL-12 were evaluated by means of ELISA. A one-way analysis of variance (ANOVA) with Tukey’s post hoc test was used. The confidence interval was established at 95% for all tests. The level of significance and *p* values are shown as * (*p* < 0.05); ** (*p* < 0.01); *** (*p* < 0.001); **** (*p* < 0.0001).

To evaluate the effect of immunization using anti-Foxp3 ASO on the production of Th1 cytokines, we measured the production of IFN-γ and IL-12 in Eno-stimulated splenocytes. These cytokines are importantly involved in the defense against *S. schenckii* [33,34]. As can be seen in Figure 7, the mouse cells immunized with the vaccine formulation containing 8 µg ASO showed greater production of these cytokines than the rest of the groups. However, when stimulation was produced with Eno + anti-Foxp3 ASO, no differences were observed in cytokine production when compared to cells stimulated in vitro with Eno alone. In this case, we expected that the reduction of Tregs would have a positive influence, causing greater production of IFN-γ and IL-12.

## 3. Discussion

In the last decade, there has been growing interest in the rational design of vaccines using defined molecules with well-characterized cellular and molecular mechanisms of action. Given the known deleterious effects of Tregs in vaccine immunogenicity and efficacy, one of the current directions of this approach is the development of subunit vaccines and molecular adjuvants targeting immune regulatory networks to improve vaccine immunogenicity [15]. The use of ASOs targeting important regulatory mechanisms is one of the most promising molecular adjuvants for vaccine improvement [16]. Several ASOs have been designed against immunomodulatory components, such as cytokines [29,30], immune checkpoints [31,32], or transcription factors [25]. Recently, indoleamine 2,3-dioxygenase (IDO), which is involved in Treg activation, has also been targeted by silencing strategies [35]. In all cases, they showed a relevant activity enhancing vaccine immunogenicity.

Foxp3 transcription is induced in Tregs by T cell receptor (TCR) signaling. Upon its expression, an autoregulatory transcriptional circuit stabilizes Foxp3 gene expression to consolidate Treg differentiation and activate the suppressive function [36]. The block of Foxp3 elicits Treg depletion and it promotes enhanced stimulation of effector immune mechanisms [37]. Foxp3 blocking has been used for vaccine improvement using different methods, including interfering with Foxp3 mRNA-transfected dendritic cells [20], synthetic peptides [22,23,24], and ASOs [25].

In this study, we used an anti-Foxp3 ASO, selected according to the results of a previous study, in which several ASOs were evaluated to improve an anti-tumor vaccine. The selected ASO showed the best Foxp3 silencing and immunostimulating activity [25]. Although several characterizations were made in that study, additional in vitro tests were included herein to deepen our understanding of its mechanisms of action. We first evaluated the capture kinetics of the ASO in murine splenocytes. This test allowed us to assess the phenomenon that occurs in vivo in the target cells. We also used two ASOs with different sizes (13 and 20 mer), and we observed that ONs in this size range can reach cells in a noticeably short time under culture conditions, barely 10 min. The highest concentrations in cells were achieved after two hours of incubation. Similar results were reported by other authors using different cells such as RAW264.7 cells [30] and lymph node cells from naïve ICR mice [31]. A parallel test was developed in human leucocytes from peripheral blood (CD45+) and the same results were observed.

The target of our anti-Foxp3 ASO was located in intron 1 of Foxp3 gene, so it is estimated that its site of action could lie at the preRNA level. In this way, the Foxp3 silencing effect and Treg depletion were evidenced in B16 cells and splenocytes, respectively. Although the microscopic images in splenocytes did not reveal the exact localization of the ONs, the heterogeneous distribution of the fluorescence suggests that they reached different intracellular regions. However, the best evidence that the ONs accessed the cell was the demonstration of the biologic effect evidenced by Foxp3 silencing and depletion of CD4 + CD25 + Foxp3 cells in vitro. In addition, under the experimental conditions used, the ONs used in this study were not cytotoxic, as previously reported [25].

The experimental vaccine used in this study was designed for the prevention of sporotrichosis, a worldwide emergent subcutaneous mycosis caused by pathogenic species of the genus *S. schenckii*. The recombinant-Enolase vaccine tested in this study was recently developed by our group and was suggested to be protective against experimental infection in mice [27,29]. Administration of anti-Foxp3 ASO as part of the vaccine formulation induced a considerable upregulation of the Th1-type cytokines IFN-γ and IL-12 in immunized mice. Moreover, it was also associated with enhanced production of specific antibodies. Interestingly, when the Tregs were quantified in the splenocytes of vaccinated mice, no differences between groups stimulated with enolase and those not stimulated were observed. The lack of differences in the presence of Tregs between the vaccinated groups may be because samples were taken 1 week after the second immunization. Thus, Treg depletion by the vaccine formulations with ASOs may not have been detected due to their being clonal and transitory. However, once again the stimulation of splenocytes with Eno plus ASO reduced the presence of Tregs in all groups.

Most of the adverse effects associated with Treg depletion occur when using products that are systematically administered [38,39,40]. Instead, the use of ASOs targeting Tregs as part of vaccine formulations could reduce off-target effects and toxicity manifestations. In this sense, the use of appropriate ASO delivery systems could help to optimize the adjuvant effect in a safer way [41]. More studies are necessary to consider the use of different delivery systems with vaccine models, including tissue distribution, pharmacokinetics, and stability analysis.

## 4. Materials and Methods

### 4.1. Oligonucleotides (ONs)

To study the Foxp3 interfering activity, ON sequences were as follows: ASO Foxp3: 5′-GGGGGAAGCACGGAAGGG′ (18 bp); scrambled: 5′-AGGAGGACAGGAGAGAGA-3′ (18 bp). The sequence of Foxp3 ASO is complementary to a region located in intron 1 preserved regions of the Foxp3 gene of *Mus musculus,* strain C57BL/6J chromosome X, GRCm38.p4 C57BL/6J (Accession NC_000086 Region: 7579676.7595243 VERSION NC_000086.7) in the US National Center for Biotechnology Information (NCBI) Nucleotide Database). Herein, we used the anti-Foxp3 2′-OMe-PS-ASO (ASO) and the control (Scrambled), as previously reported [25]. All these nucleic acids were purchased from Integrated DNA Technologies (Coralville, IA USA).

### 4.2. ON Uptake Kinetics in Murine Splenocytes

Spleens from 12-week-old male C57BL6 mice were aseptically removed and a suspension of splenocytes was prepared as previously described [42]. Two oligonucleotides (ONs) of 13- and 20-mer sizes with phosphorothioated, backbone labeled with either cyanine-5 (Cy-5) or fluorescein-5-isothiocyanate (FITC), and were used to evaluate the ON uptake kinetics in murine splenocytes. Labeled ONs of either 13 or 20 mer were incubated independently at concentrations of 0.5, 1, 2, and 4 µM at 37 °C for 0, 10, 30, 60, and 120 min with a of 1 × 10^7^ cells/mL suspension of splenocytes in RPMI 1640 medium (Merck KGaA, Darmstadt, Germany), supplemented with 10% Fetal Bovine Serum. At each time and for each cell concentration, 50 µL of cell suspension were taken, properly dissolved in phosphate buffer saline (PBS pH 7.4), and analyzed by means of flow cytometry to measure the relative fluorescence (as RFU). A Gallios cytometer (Beckman Coulter, Brea, CA, USA) was used in all analyses.

Several samples of splenocytes treated with Cy5-labeled ONs and incubated for 1 h at 4 µM, from the experiment above, were also marked with anti-mouse CD4 FITC antibody (RM4–5) (Thermofisher Scientific, Waltham, MA, USA) and fixed with 4% formalin. Images of labeled cells were acquired by the high-content screening (HCS) InCell analyzer 2200 system (Cytiva, UK) to observe the uptake of ONs in CD4+ lymphocytes.

### 4.3. Biological Activity of the ASO Foxp3

The silencing effect of the anti-foxp3 ASO was confirmed in the murine melanoma cell line B16, which expresses high concentrations of this transcription factor. These cells are syngeneic with the C57BL/6 mice (Charles River Laboratories, Wilmington, MA, USA) employed. Cells were incubated with 2 µM ASO for 48 h. To quantify the Foxp3 mRNA, RNA was extracted from cultured cells using a commercial kit (NuceloSpin^®^ RNA-Blood, (Machery-Nagel, Bethlehem, PA, USA) according to the manufacturer’s instructions. RNA was retrotranscribed into complementary DNA (cDNA) using random hexamers and the enzyme reverse transcriptase High capacity cDNA reverse transcription kit, (Applied Biosystems, Foster City, CA, USA). The mRNA copy number of the gene was quantified by means of quantitative real time PCR using the 7900HT Fast Real-Time PCR System (Applied Biosystems, Foster City, CA, USA). TaqMan probes to amplify Foxp3 were obtained from Applied Biosystems (Foster City, CA, USA). The silencing efficiency was obtained by comparing the number of mRNA copies of these genes in the treated groups with cells treated with a respective control (scrambled), plotting the results on a standard curve prepared with a known amount of Foxp3 copy number.

The activity on Treg depletion was evaluated in splenocytes cultured with 2 µM of anti-Foxp3 ASO or a respective control scrambled ON for 48 h. The presence of CD4+ CD25+ Foxp3+ lymphocytes was quantified by means of flow cytometry using the eBioscience™ Mouse Regulatory T Cell Staining Kit #3 (Thermo Fisher Scientific, Waltham, MA, USA).

### 4.4. Recombinant Sporothrix schenckii Enolase

Recombinant *S. schenckii* enolase (Eno) used as the antigen in this study was obtained and characterized as previously described [27].

### 4.5. Cytotoxicity of ASO Foxp3 and Eno

The cytotoxicity of Eno (10 µg/mL) alone or in combination with the anti-Foxp3 or the scrambled control ASO (2 µM) was evaluated after 48 h incubation. These concentrations were the same as those used in the following studies. Cell viability was analyzed by flow cytometry using the combination of PI/Annexin V-FITC (Thermo Fisher Scientific, Waltham, MA, USA) to determine the presence of necrosis (PI+ Annexin V−), late apoptosis (PI+ Annexin V+), early apoptosis (PI− Annexin V+), and living cells (PI− Annexin V−).

### 4.6. Adjuvants and Vaccine Formulation

The vaccine formulations were prepared as follows (Table 1):

### 4.7. Immunization Schedule

Male C57BL6 mice (*n* = 7) between 6 and 8 weeks of age received the subcutaneous (sc) administration of the vaccine (on days 0 for priming and 14 for boosting) on the back of the neck, with one of the vaccine formulations described above. One week after boosting, mice were sacrificed under anesthesia and bled by cardiac puncture to obtain serum, which was aliquoted and stored at −20 °C until use. The experimental procedure (Code: 2019/VSC/PEA/0279, 15 January 2020) was approved by the Biological Research Committee of the University of Valencia, Spain) and followed the European and Spanish directives for animal care 63/2010 and RD 53/2013, respectively.

### 4.8. Quantification of Anti-Eno Antibody Response by ELISA

The titration of IgG, IgG1, and IgG2A types of anti-Eno antibodies was carried out as described previously [27]. Briefly, a 96-well ELISA plate (Costar^TM^, Thermo Fisher Scientific, Waltham, MA, USA) was coated with 5 µg Eno/mL in PBS at 4 °C (overnight). The plate was washed with washing buffer (0.1% Tween 20) and then blocked with 1% PBS-BSA for 1 h at room temperature and washed again. Specific IgG antibodies (total and 1 and 2A subclasses) against *S. schenckii* Eno, induced by the vaccine, were made in the serum of the vaccinated animals and controls. Serum samples were diluted in PBS-BSA 1% -Tween 20 (0.1%), at a 1/1000 ratio to determine total IgG and the IgG1 subclass and 1/100 for the IgG2a subclass, and were added to the ELISA plate. These samples were incubated at room temperature for 3 h. Antibodies were detected with total goat anti-IgG (Biocheck, South San Francisco, CA, USA) at 1/10,000 dilution and with anti-mouse IgG subclasses at 1/1000 dilution (Mouse monoclonal isotyping reagents; Sigma-Aldrich, St. Louis. MO, USA), followed by a 1/5000 dilution of biotinylated rabbit anti-goat IgG (Sigma, Sigma-Aldrich, St. Louis. MO, USA) and streptavidin coupled to horseradish peroxidase (Merck KGaA, Darmstadt, Germany). The plates were finally developed with a mixture of 30 mg/mL orthophenylenediamine (OPD) (Merck KGaA, Darmstadt, Germany) and hydrogen peroxide. The reaction was stopped with 1 N HCl to read the absorbance at 492 nm.

### 4.9. Quantification of IFN-γ and IL-12 in Splenocyte Culture Supernatant

Splenocytes from immunized and non-immunized mice were cultured as described above and stimulated with 10 µg/mL Eno or 10 µg/mL Eno+ 2 µM of ASO for 24 h. The levels of both IFN-**γ** and IL-12 were measured by means of ELISA in the culture supernatant, after stimulation, according to the manufacturer’s instructions (Pharmingen, BD Biosciences, Diego, CA, USA).

### 4.10. Statistical Analysis

Statistical analysis was performed with Prism software ver. 6.01 (GraphPad, San Diego, CA, USA). One-way analysis of variance (ANOVA) with Tukey’s test of comparisons was used. The confidence interval was established at 95% for all tests. The level of significance and the *p* values are shown as * (*p* < 0.05); ** (*p* < 0.01); *** (*p* < 0.001); **** (*p* < 0.0001).

## 5. Conclusions

In summary, the Foxp3 ASO used in this study is a safe and stable molecule that is suitable for improving the immunogenicity of adjuvanted vaccines as part of the vaccine formulation. Treg depletion seems to be the main mechanism of immunostimulation. Future studies will contribute to unraveling other mechanisms of ASO anti-Foxp3-induced immunostimulation and its safety profile. Another issue that is being evaluated is whether ASO can act alone as a vaccine adjuvant in different formulations and by different routes of administration.

## Figures and Tables

**Table 1:** Vaccines composition.

Vaccine Formulations/100 µL/Mouse
PBS (Control)
100 µg of Eno in PBS
100 µg of Eno in PBS + 5% Montanide Gel 01 adjuvant (Gel 01) kindly provided by Seppic (Paris, France).
100 µg of Eno in PBS + 5% Gel 01 + ASO anti-Foxp3, 1 µg
100 µg of Eno in PBS + 5% Gel 01 + ASO anti-Foxp3, 8 µg.

## Data Availability

No new data were created or analyzed in this study. Data sharing is not applicable to this article.

## Supplementary Materials

The following are available online at https://www.mdpi.com/article/10.3390/ijms22073470/s1.
