# Supplementary material for: Foxp3 Silencing with Antisense Oligonucleotide Improves Immunogenicity of an Adjuvanted Recombinant Vaccine against Sporothrix schenckii
Source: Int J Mol Sci. 2021 Mar 27;22(7):3470. doi: 10.3390/ijms22073470 (PMC8037512; doi:10.3390/ijms22073470)
Supplement: Supplementary file 1 [file ijms-22-03470-s001.pdf]

>NC_000086.7:7579676-7595243

AGTTTCCCACAAGCCAGGCTGATCCCCCTCTAGCAGTCCACTTCACCAAGGTGAGCGAGTGTCCCTGCTCTCCCCCACCAGACACAGC
TCTGCTGGCGAAAGTGGCAGAGAGGTATTGAGGGTGGGTGTCAGGAGCCCACCAGTACAGCTGGAAACACCCAGCCACTCCAGGTA
AGGACTTTGGAAACTAATACCATTCATCCTAAATGCCAGATAGGTGGAGCAGTTGGTCCTTAGACAGGGGCAAAAAGAAGTACTTTG
ATTGTTTGATGCACAGATAAACAGGATTTTTTTTAACATATGTCTATCAACTGCTGGTCTCCAGGAATGCCGGAGCTTTAGGCAACTC
AAGATGCTGTCCAGCTATAACTAGAAACTAGAAGTGCATCTCTTGTTTCTTTTCCTCCTGCTGTCTTCCATTTCTTCTCCTGTCTCCCCCT
GCTTCTTTGCCTGTCTCTGCCTTCCATCAGTGCCCAGTCTCTGTCTCTTTCCCAGGCTCTGACTATATGCCTGTCTGTCTCTTCCAGCCTG
GCCAGCCACAGTCCCTTTCTTTCCTCCCGCTCTCTGACTCTCGGCTCATCTTCCTTCAGCTGCTTTTGCACCCGGTATTGAGCGCAGATA
TTTGTACACAACTGGCGCTTAATAATAATGGCTTAAGAGCTCTGTTTTCCAAGAACGGGCATTAGTTCTGTGTGTCTTAGGTTTGTGAG
CTGTCAGGTCAGTCTTAGCATTTAACTGACCTTCTGCTTGTGTCACGCAAGATAACACCCTCAGTCAGCCACAGTTTAGCAAAGGACT
ATATGACTGTGAGCAGAATCCATGTGCAAGGAGAGCAGGCAGTTCAGGACGAGGGTGAGCTGGTCTCTGCAGGTTTAGTGCTGTGGC
ACTGTGCCTGGTATATGGTGAGTTCTCACTGTTTGCTATTAGCATTTTTAAACAAATTAGAATCGTGCTATAGATTGGATTTGTTTCTGC
TCTGTTCAAGCGATACCATTTTTGTAGCATACTAAAATAACGAACACGTGATCTTTTATGTCTGCCGTGACTGTCCTCACATCACCATG
AATTTGATTACCTGAATTAAGTGCTGATGGTGGGATATTTGGGTTTCTTCTGATTCTAAAACTCCATACCTGACTCCATGGATCCTGAA
AATGGAGTAGCTGGGGAAGAAGGGGTGTACATCTTAAGGGGTTTTGCCCTCTCTACAAATTGCTTTTCCAAAACGTTGTCTTATTTTCT
GTTGTTTCTATTCAAGTTAATTTTAGGTGTGTGTTACCATTTTTAATCCTTCCCCATCATAAGAAAAATGACAAGTATTAAAATTTTGCA
TTTTGGTTACTTTTAATGACCACTGACCATTTGTGCTCTGTAGGCTAGAGTTTTATGATCACATTCTTCATCCTACTAAATTGCTCATGAT
TTTTCAAAATTGCTAATAGCTCTTAATTTAGAAAGGATAATAACTATTGGCTATATGTATATGACACATATTTCCCTGAAATTCATCAT
TTGTGTGTATGTGTATTTTAATTGTATTCATTTACTTAGTTTATGAGCATGCATGTTCTTCCTGCATGTGCACCATATGTGTGCCTGGTGC
CCACAGAGGCCAGAAGATGGTGTGGGATCTCATGGGACTGGAGTTACAGATGGTTACGTGGGTGCTGGCGCTTATGTGGCTTCTTTCT
ATGGTTTTGTGTTTAAAAGCCTTTTACCACTTGAAAATGAGAAGCTACCTCCTCTACAAGAGCAGCAGTGCTCTTACCCATGGAGCCA
TCTCTCCAGCCCTATTTGTATGGGGGGGGGGGGTCTTCTGAGACAAGGTCTCACTCTATAGCCCTGACTGGCCTAAAACTCACTGTAT
AGACCAGGCTGACCTCAAACTCACAAAGACCCATCCATCTGCCTCTGCCTTCTGAGAAGTGGGATAGAAGACATACACCACCACGGC
GGGCAATCACTTGCTTTTTTTCCCTATTTATTGTGCTTTGTAATGCATGTGTCTTTTAGGTCTTTAGATTACTCTTTTCTTGTGGGGCTTCT
GTGTATGGTTTTGTGTTTTAAGTCTTTTGCACTTGAAAATGAGATAACTGTTCACCCCATGTTGGCTTCCAGTCTCCTTTATGGCTTCATT
TTTTCCATTTACTGCAGAGGTCAAAAGTGTGGGTATGGGAGCCAGACTGTCTGGAACAACCTAGCCTCAACTCAAGTCATCTGTGTGA
ATTTTACCCAGGCTCTTAACCTCTCTGTACCTCCATTTCCTCGTATGTACTGTGATGATTATAACAGTACCTACCTCAGAGGATCTTTCT
GAGGATTATTTTTATTAATGATGGTAGGTGCTCAGCACAAGGCCAAACAACAATGATAGACATTAAAACGTATCTCTCTAGTGGGTCT
GGAAATTATTCTAGAGCGTCTGATGACAGCGACATTTCAAGTGGGCAGGGAGGTATTGGTGGGAAAGTGGGCTATCTACCCAGTCAC
TTTATTTTCCCCTAATTGTCTCAGAATCATTTGTTAATCTGTCCTGCACTGTTCCTCATGTTGAAATGTTGTGTTCATCACAAATTCCATT
CCCTCTGTGCATGGGTCTCTGCCACGGTTTTCTACTCTAATCTGCTCCTTAGTGTTTATTCTTGTACAAAGCCCACACTATTTTTCTGATG
TTGCTTTGCAAAACAATTCAATACCAGCCATGGGTGTCTCTGGCACCTAGCAGCATCAGTCCTCCAGCCAGAGGCCAGTGATTATTTT
CAGTCCTTTCTCTCACTCCCTCTCTCTCTGTCTCTGCATGTCTGTCTGTCTGTATATGTCTCTGTCTTGTTCATTCTTTCTCTGTCACTTTTC
CTCTAAACTGCTCTCACTGTCTCTCTCTATGAGCTTGATTCCTATTCCATCTCATGTTTCTCTCTATATATTTCTCTATCTGTATCTCTTCT
ATATCTGTATTCACACACATATGATATATATATATATATCTCAATATATATATATATATATATATATATATATATATATATATATATATA
TATATCAATATATATATCTCATACCATACCATACATACATACGGCTATATAGCTCCATAAGATTTACCCCAGCCACGAGACAGAAAG
ATGCTGGCCTTCCTCCACCTCGTACTCTTCCCTCCCCAGTCTAGAAGGGCAAACTGGGCTCAGAGATGAGCAGCCCCCACCCCCAGGC
CTCACAGAGATGTTGTGTCAGAGTTAAATCCAAGAGCAGATCTCAGAATTCTCAGTGGGACCTTGACTTTGGCAATTCCACATTGCAG
GCCTTAGTTTACCTCTCAGGACCCAGGAGGCCATTAACAGGAGACCTGAGGTGCCCTTCCCTCTTCTACATCCTCATGAGTTGGATCC
AGTCCATAACCATAGCATGGGGCCAAATCTCACAAGCTCTGGTCTATGTGAGGTTCTGGGCCCCATGAGTCAGAAGTCCTAGCGGAC
CAAAGAACACTAGTAACGATGGAGAAATATCAGTTAAGTATGAACCCTCAGAGTTCATACTGCATTCCTTGGGACAACCATTCTGGG
GCCCTTCCAAAAAGCCTGGTGGTGTGCTCTTTCCATGAGGGCCAGGCCAAATGTCTTCTTCCTCTTGTCCCTGTATCTGGAAGAATGTT

ATAATTTGGGGAAAGTTGTCCCAGGAGAGCGGGTCTGGAGCCATATGTAAGTGACCATTTATCAGTCATAGACACTTGCTCAGCATTC
TGTATGTACGAACTTTGCAAGATGGCTCCTGTTACTGTCCCAAATTAGACAGGAGGACAGAAAGACCCCAGCCTTCCTCCATCACATA
CTCTTCCCAGTCTAGAAGGGCAAACTGGGCTCAGAGATGGACAGGAAGGCCCCTTTGTCCCAAGAGGGCAAAGCCTGACCCCAGAT
CAGGACAGTAGAGGGTTTTCCAATCCTCTGTCATAATGGAGCTCAGGAGGGAGGGAGGCTGACATTCCAGAGCCAGCAAGAGGCCTT
ATGGAGTTTTAAGCTTCCTGGCTTTAGGTGGTTCCCATTTCTTTGGGCTCTGGGACATCAATACACACAGTAAGAAGGTGGATCCATGC
ACCCTACAGAGTCTGTGTTCTTGAGATTCTAAAATCCGTTGGCTTTGAGAAATGATATCGTACAGTTCTGAGTTTCTGTTACTACAGCA
TTTGAAGACTCAAGGGGGTCTCAATATCCATGAGGCCTGCCTAATACTCACCAAGCATCCAACCTTGGGCCCCTCTGGCATCCAAGA
AAGACAGAATCGATAGAACTTGGGTTTTGCATGGTAGCCAGATGGACGTCACCTACCACATCCGCTAGCACCCACATCACCCTACCT
GGGCCTATCCGGCTACAGGATAGACTAGCCACTTCTCGGAACGAAACCTGTGGGGTAGATTATCTGCCCCCTTCTCTTCCTCCTTGTTG
CCGATGAAGCCCAATGCATCCGGCCGCCATGACGTCAATGGCAGAAAAATCTGGCCAAGTTCAGGTTGTGACAACAGGGCCCAGAT
GTAGACCCCGATAGGAAAACATATTCTATGTCCCAGAAACAACCTCCATACAGCTTCTAAGAAACAGTCAAACAGGAACGCCCCAA
CAGACAGTGCAGGAAGCTGGCTGGCCAGCCCAGCCCTCCAGGTCCCTAGTACCACTAGACAGACCATATCCAATTCAGGTCCTCTTT
CTGAGAATGTACTGATGCATCACACAGTCACACCAGTTCCACAAGTATTTAAGGAGGAGATTTCTTATAAGTTCTGACCAAACATAA
AGAGCACTTCAAAAGTGACCATGGTCCAGCCATATGGGTTAAGCCAATATAGTGGAAAATTCTACTCACCAAACCTGATCCGCATTT
GCTTGAGCTACTGTAATGAAGTATCACAAACTGGGGGACTTACATAGCATAGAATTATCATGTTAGCGTTCTGGAGGCTATAAGACCA
AGATGAAGACGTCAGCAGGGTTGATTCCTCCTGTAAGTCCTGGCCTCCTTCTCATCTCTGATGCTTTCCTTTGCTGTTCTTTCTTGGAGG
AGCATCACCTCATGGCTGCCTGCCTGCAGTCTTTCAGCTCATCGCATCACGGTTCTAGGAAGCCAGTCTCAGCTTCCACAGACCCAGA
CTCCTCTTTTCATGCTAATGTTTTAGCCCGTGACACACTAGTCTTAATACCTAGGTTCTCATATAAATCTCTCAACTCTGATAAGCCCCA
GACATGATAGCAAAGAAGATGCAATTGCCTTCCAAAACCCTTCCGTGCTTCCCCCAGGCTGTTCTCAGAAGCTACATGCCCAACACAT
GTAGTATATAGTAGAACGGAGAATGACATATTCACATGCACACACAAACACAGCAGGGAAAATGTACATATATATACTTCCTAGAG
AAAAATGAGGCAGTATCAGCCTGAAATGGTGGTTTATAATCCCAGTACTCAGAATGCAGAAACAAGGAGTTCAAGGACAGCCTGGG
TATATAAGGAGTTCCAGACTACAAGAAACCCTATCTAAAAAGAAAAGGAGGTCCCAGGCCATGAGAAGACTATAGAATTCTGAACC
TGGCTATCCTCTTAATTAAAATCAGGGTAGAATTCTATAGTCAGTTCAAGATCTGGTTCCCTCTCTGACTGGAAGTATAGGATCCTGAA
AAACGAAAGCCACACTTTTAAGGGACTGTAAGGTAGTGAGGCTCAGCACAGGGACCTGGGTCACCATGTAGAGCTTTGAAGAGGAA
ATCAGAAGACTGCAGTATGGCTAAGGGAAGAAGTGGACTTCCAAGCTTGGCAGAGATTGGAGCTAGTTTGAGGAGCGCCCAGGGAC
CCTCAATCAAGCAACCCTATCCCTCTTTTTTTCCTGGCACCTGCCACGCCAATTCCAAGACAGAAGAAAGCTTAGAGAAGACAGACC
CATGCTGTGGCCCTGAGCTCTGCAGTACTGAATTCAGCTGCAAGTCTTCCCTGCCTCTACTGCTTACCTTTGCATTTAGCCACATCTGAC
TATCACTGTATACTCTGCTCCTCCATCCTCTACCCTCCATCTCCAGTAATGCTCCTGTTGTAGCTGCTTCTGCCAAAAACCTAGACATCA
TCTTGACCCTTTCTCTCATCTCCTCCATCCAAGCTCCCGGCAACTTCTCCTGACTCTGCCTTCAGACGAGACTTGGAAGACAGTCACAT
CTCAGCAGCTCCTCTGCCGTTATCCAGGTTGGTAGCAGCAACACCACTCGCCTCACTATTGCAGTACACTTCCCACTAGCACAGTTCC
CTGGAGCCTTCCTGCTCACAGCATCCAACTGAATCTTGTGAGGCTATGCCCAAGTCATTGGAATAAAAAGATGAGAAGAGAGTCCAA
GACAAGCCCCAGTAGAATCAGCAAAGACTATGTGGCCTGCACAGAGTGCAGGGGGTACTGGAGGGTCCCACAAACCAACTCCCCAT
CACCCCACATTCACGACAGAGTGGTATGGTGTATGTAAGCAAGTGAGGTGCTGGACATGTGCATGTGTAGAATATATCCATCAATCTG
TGTTCCTGCTGTCAGGGTAGCATATATGTATGTAAGACAGACCAGAGGTGTAGTTATGAGGCTATCTTGCACCACCCCTGGAATGCAT
GTGACTCCATTCCACTGTTATCCCTGCAGCCTGCCTCTGACAAGAACCCAATGCCCAACCCTAGGCCAGCCAAGCCTATGGCTCCTTC
CTTGGCCCTTGGCCCATCCCCAGGAGTCTTGCCAAGCTGGAAGACTGCACCCAAGGGCTCAGAACTTCTAGGGACCAGGGGCTCTGG
GGGACCCTTCCAAGGTCGGGACCTGCGAAGTGGGGCCCACACCTCTTCTTCCTTGAACCCCCTGCCACCATCCCAGCTGCAGGTGAG
GCCCGGGGCCCAGAATGGGGTAAGCAGGGTGGGGTACTTGGGCCTATAGGTGTCGACCTTTACTGTGGCATGTGGCGGGGGGGGGG
GGGGGGCTGGGGCACAGGAAGTGGTTTATGGGTCCCAGGCAAGTCTGACTTATGCAGATATTGCAGGGCCAAGAAAATCCCCACTCT
CCAGGCTTCAGAGATTCAAGGCTTTCCCCACCCCTCCCAATCCTCATCCCGATAGGAGACCTTATGATTCCATGGACATAGCCATGTA
TCCTCATCCCACTGTGACGAGATGGCTGGGGCCCAAGAAGGTAACAGTGTTGGGGCCAGCTCTACCCCTTGAAACTGTTGGACCTTGA
TACATTCACTCTCCACGAGCCTCAGATTCCACTGATGTGAACTGGATAGTTCCATTGTTGCTACCGTGTGAGACTTTAGTAAAGAGCTA
ATGAATGAGACACAGAACTATTAAGATGAGGCTCATGGCATCTCATGGCATCTCCCTTCTCTCTCCAGCTGCCTACAGTGCCCCTAGT
CATGGTGGCACCGTCTGGGGCCCGACTAGGTCCCTCACCCCACCTACAGGCCCTTCTCCAGGACAGACCACACTTCATGCATCAGGT

ATGGAATCGGAGCAGGCTGGGAGGAGGGAACAAAGAGGACAGCTGTGGAGCAGAGCCCCAAGCCCCGCTGAGCCATGGTCCATGT
GTTCCCCAGCTCTCCACTGTGGATGCCCATGCCCAGACCCCTGTGCTCCAAGTGCGTCCACTGGACAACCCAGCCATGATCAGCCTCC
CACCACCTTCTGCTGCCACTGGGGTCTTCTCCCTCAAGGCCCGGCCTGGCCTGCCACCTGGTAACACCTTCACAGTATCTCCAAGTTCT
CTAATCTTTGAGCATGTGCAATGTAAACTTTTCTGAATTATAGCCCTATGGAGGTATAGAAGGGTCTTAAGAGTCACGGAAACTCCAA
CCTCCAAAAAAAAAAAATATCAGACTTAGAACCTTGAAGACATAGAATGCAAAAAAAACCACAAATCGCTATTATCAGTCAAAATGC
CATCACTTACCAATGGGCATCTTTAGGCTGTTATGTCAGAAGCCCTTGACTGTGGGAACAGCAGAGTACTATGAGACAGAGTCTTCAA
GGCTCAGGAAGGGGAGGGGCCTTCTGGAACAAGCTGTAGAGTCTAACCTGCAGCTCCAGAAGTACCCTGTCTCTACCCACAGGGATC
AATGTGGCCAGTCTGGAATGGGTGTCCAGGGAGCCAGCTCTACTCTGCACCTTCCCACGCTCGGGTACACCCAGGAAAGACAGGTGA
GTTGGCAGGGCTGGCAAGAAACGGCCCCTGCCCACACCTCACCCCACCCCTGCACCTATTCCTCTGCTGACATCCCATATTCTCCCAT
CCCCAGCAACCTTTTGGCTGCACCCCAAGGATCCTACCCACTGCTGGCAAATGGAGTCTGCAAGTGGCCTGGTTGTGAGAAGGTCTTC
GAGGAGCCAGAAGAGTTTCTCAAGTGAGTAGCCTGACCCTACCCACAGAGTTCTGCTGTCTAGGCTTCACGTCTCAACTCACCATCCT
CTCAATGGATGATAATAAGAATCATAAAGATTCAGACTCCATCCCTCCCTGGCTCTGTGATCTTGGGCAAGTTATGGGTCTCTAGGCC
CAGTTTACCTCGCATGTATGAAGAGACATAATAATAAAGGTATGTGCTCATAGTTACCTTCCTGTTACACGCAGAAGGATCTAAGGCC
ACAGAGAATTAAGGGTCAATCAAGCTCACACAGGACCTAAGTGATGAATCTTGAATATGAACACAGGCAGCCAGGTTCCAGAGCCC
ACACGCCTAACTGCTTTGTCCCGCTTCCCCTCACACAAAACACATTCCTGATCCTCCAATTTCTGTTCCTCTAGATGACTATAGAGCTC
TTGCCTCTCTGCTCTCTATCTGCTGTCCCTCCCCTTCTGTATCTTGCTAGTCACCCCTAACTTTTGGCAATGGTGCGTGTTTGCGTGGCCA
GGCCTTTGCATGGGCTGTGCCTGACACCTGAAATGCCATACCCCTGCATACCTCCTGTCTAACGTCATCCCAGCATTTTGGCCAGACTC
AAAGGGTAAATAAGCTCAGGCCTGGCAGCCCAGAGTTGCTGAAGCACATGTGTTTAAGGCAAGCAAGGGGGTGGGGGGGGGAGCA
CTGAGCATAGAGAAATCTCCCAAAGGGTCTAGGCCGTCCCTAACTGATACACTAAGCCAAGAGGCCTGACCCACCATGGTCAGCTAC
ATGGAATCTTCTCCTTACTCAGGCACTGCCAAGCAGATCATCTCCTGGATGAGAAAGGCAAGGCCCAGTGCCTCCTCCAGAGAGAAG
TGGTGCAGTCTCTGGAGCAGCAGGTAATGCCTGCAGGGTGTGGCTGCGGGGTGTGGCTGCGGGAAAGAAGGATGGGAGGGAGGACC
CTGTGAGGGAAGGCATGGGCAAAAGTGTGCCTGAGAACGACCAGGTGGAAGCCCCACTTTGGTGTACATCCCCACAGCTGGAGCTG
GAAAAGGAGAAGCTGGGAGCTATGCAGGCCCACCTGGCTGGGAAGATGGCGCTGGCCAAGGCTCCATCTGTGGTGAGTACCCCAAG
TCCAGAGGCAGCAGACTTCAACTGCTGAGGGGCAAGACAGGAGCCCATAAGGACCAAATGTCTTCTTCTCACATGCAAGCCCTGCCC
TGTACAGACCATTCCCACCTAATTAATATGCCAGATCCAAAGACACGCCTACTCTGCTTACAAACCTTCTGACCTCCAAAACATTATG
ATTCTGCCTTTTCAGGGCACATACAGAAGGCAGTGAACTCACAGGGCCACTGCAAAAAAGGAAAATGGAGGGCCTTATGTTCAAATT
TCAAGATAAGCTCAGAACATCGAACAGTGTGTGACCACACATTTCACATACCCAGTCTCAGGCTGATATGAGTCTTATACTATAACAG
AGGTAGCTACCACCATCATCCTAATGCACAAATGAGGACAACTTAGGTCAGGAAGATTTAGTTGATGCTCCCAGGTTCACAGTTGGT
GCTAGGGGATTCCAATTCTGCCCCTGCTCACCCCAGCCCTAGCATCTATGGCTTCATCGCATGCTCATGCCTGTACTCTAAGATGCTGC
TTTACAGAGCTCCACCAGAGCCTGCAATTGACTATAGGGTGGTGCCCTTCTCAAAAGCATTGACCTTACTGGACACAGTGGCATGCAC
CTGTAGTCCTGGCTACTGGAGAGGCTGAAGGAGGAGCACTTGAACCCTCAAGTTCAAAACCAGCCTGGTCAACACAGAGACACCCT
GACTCTTCTAAAACACAAAGAAACACGGTTGGGGAGAAACTTGAGAGGGAAAAGTGATTGCCATACAAGGATAAGGACCTGAGTTT
TGCTGGGTGGTGGTGGCGGCGGCGCATGCCTTTGATCCCAGCACTTGGGAGGCAGAGGCAGGTGGATCTCTGTGAGTTGGAAGCCAG
CCTGGTCTATAAAGCTAGTTCCAGAACAGCCAGAGCTACACGGAGAAACCCTGTCTTGAACACCTCTGACAGAAAAAGGACCTGAGT
TTAGATGCCAGCACCCACACCAGATGCAGCACTGTAAATCTGTAATCCCAGCATGTGTACACACACCACACATACAAATCAGATAGA
AATATGACCAAATCAGGAAATGCAAATTGTAAAATAAAGTGGGGTTGGGGAACTGGACAGATAGCTCAGGGATTAAGAGAGCTTGC
TGCTCTTTCAGGGGACCAGAGTTTGGTTCCCAGCACCCTCAGAGCCGCTCACAGCTATCTCTAACTCCAGTTCCAGTGGATCCAATGC
ACTTTTCTGCCTTCCACAGGTACCAGGCACACATGCGATGCCCAGACATGCATGCAGGCAAAACTCCCGTATACCTAAAATAAAATG
CAAGCTGACTTGGCAGTAATCTCAGCCCATCCTGTGCTACATAGTACATGTTAGACTAGCCTGTACTACATGCTACATAGTACATGTT
AGACTAGCCTGTACTACATGCTACATAGTACATGTTAGACTAGCCTGTACTACAGAGCAAGAGCCCACCTACATAAATATCCAACCA
AGCAAGCAATCATTTTTTAAAGTAAAATGGAAGACTCAGTGTGGTGGCGCACGCACGCCTTTAATCCTAGAACTCGGGAGGCAGATG
CAGGCAGATCTCTGTGAGTTCGAAGCCAGTCTGGTCTACAGAGCCTGGTCTATACACTGAGCTCCAGGACAGCCAAGACTACACAGA
GAAACCCTGTCTGGAAGAAAAAAAAAAATATATATATATATATATATATATATATACATAAAATAAAAAGTGGAAGCCAGATGTGGTGG
CACACACTTATAATCCTAGCACTCCAGAGGTAGAACTAGGCTAGAAGGTGCAAGGCCAACTAGAGATATATAGTGAGACTGTCTCAG

ACAAAACGAAAATGAATAGGCAAACACTCAGGAGGCAGAGGAAGTGCATCTCTGAGAGCTGCAGGCCAGTCAGGGCTACATAGTA
AGACCCTGTCAATAATAATAATAATGGCAATAATAATTTTAAGACCAAAATAAATAGACATGGATGAAGGGGGAAAGGAATGAGAA
GAAGGAAGATAAGCGATGAGGGAGGAGATAGGGTGAAAGTGGTCTGTATGTATTACATACATGTACAAAATTGTCTAAAAACAAGT
TTAACTAATAAGAAAATACAAACTAATGTTTGAAAGGCTACAATGAAATGACAAGCTTAAGTGTCTCGATTACCACACCCCTCCCAA
CCCCTCAGGCCTCAATGGACAAGAGCTCTTGCTGCATCGTAGCCACCAGTACTCAGGGCAGTGTGCTCCCGGCCTGGTCTGCTCCTCG
GGAGGCTCCAGACGGCGGCCTGTTTGCAGTGCGGAGGCACCTCTGGGGAAGCCATGGCAATAGTTCCTTCCCAGGTCAGTGGAGTCC
ACACCCCAGTGCCAGGGGGTACAAAGGAGCTCCCCCACCCCCCTCACCCCCACTAAGAGCTGGGAGGAAACTGCACCTGAGTTTATT
AGGCTTAGAAGCCCTCAACTGTTATAAATGCATAGCCTTGGGCCCCGTGTTTTGGGGGATTGGAGCCAGGCCTGACCTATTTGGCATC
TGCTACTTCATTCAGTCACCATGAGGGAGGAGCCTGGCCAAGTGAGTCCAAAGAGCCCTCTCTTCCGTCCCCACCTCCAGGAAGTCA
GGTGCACTCAACCAAGCTAACCAACCCTCTCCCACCTGTCAGGCCTGGGTTGTGAGTTTACCAGGGACCATAGATATTTGGTGTCAGG
CTGGCTATGCCACTTGAGCTGCTTACATGCCTTTGATGTACAAATTACTTGACTCCTTTTTAAAGTGAGGAGAGCTATTTGGCAGGAGT
ACTGCAAAGAAGACACAGCTTACGGCGGGTACTCAGTAAACAGTACTATGTGTGAGCATAGACTGTCCCTCCCCCCTTGGTGCTAGT
GGTAGGAATTGAGACCTTGGATTCCTGATGCAGACAAAGGTGGGGTAGGGGGTGAGGAGGCCAAAGGCTCTGATCTATGCCAACCTT
CTGCAGAGTTCTTCCACAACATGGACTACTTCAAGTACCACAATATGCGACCCCCTTTCACCTATGCCACCCTTATCCGATGGGTAAG
CAGGGCAATAGAGGCCCAGCAGCTGGTGGGCGGCAGGGGGGGAGTTGTGGTGGGGAGTGCTTGCCTCCTACATTGCACCAAGAGCA
GAATTCACCCATTAACAAACCTCAGCTCTGAGGAGCCCCAAGATGTGATCCTTCTTGATAGCTTCACCTCAGATCTAGCCCTCAACCC
AAAACTACTGCAAGCCAGGTCAGTGCAAAGCAAACTGTAACACTACAAACTACCCTTTCCTTTGTCCACCCTATCTCTAACATCACCC
TTGACCTCATGCCTCACCCTATTCTTTCTCCTTCCCCTTGACCCACAATTACAAAGCTATCATAGCTCAGAGGGCCGAGAGTAGGCTGC
TCCCTCAGCCACAACCCTGAGGAACATGCCCCTTATTCCACCTGACTCCAACTTCCAGGCCATCCTGGAAGCCCCGGAGAGGCAGAG
GACACTCAATGAAATCTACCATTGGTTTACTCGCATGTTCGCCTACTTCAGAAACCACCCCGCCACCTGGAAGGTGAGTTCCTCTGTA
CACACTGGCAGCTGGGATGGCTCCAAGGATGGTTAGCCTGGGGCTAGACATGTGGGGAAGGAGCAGGTCAGTCTCAGACTCAGGAT
GACTGTCAACCCTGTCCCTGACTGGGGTCCCGGTCCCCCTTCCACAGAATGCCATCCGCCACAACCTGAGCCTGCACAAGTGCTTTGT
GCGAGTGGAGAGCGAGAAGGGAGCAGTGTGGACCGTAGATGAATTTGAGTTTCGCAAGAAGAGGAGCCAACGCCCCAACAAGTGC
TCCAATCCCTGCCCTTGACCTCAAAACCAAGAAAAGGTGGGCGGGGGAGGGGGCCAAAACCATGAGACTGAGGCTGTGGGGGCAA
GGAGGCAAGTCCTACGTGTACCTATGGAAACCGGGCGATGATGTGCCTGCTATCAGGGCCTCTGCTCCCTATCTAGCTGCCCTCCTAG
ATCATATCATCTGCCTTACAGCTGAGAGGGGTGCCAATCCCAGCCTAGCCCCTAGTTCCAACCTAGCCCCAAGATGAACTTTCCAGTC
AAAGAGCCCTCACAACCAGCTATACATATCTGCCTTGGCCACTGCCAAGCAGAAAGATGACAGACACCATCCTAATATTTACTCAAC
CCAAACCCTAAAACATGAAGAGCCTGCCTTGGTACATTCGTGAACTTTCAAAGTTAGTCATGCAGTCACACATGACTGCAGTCCTACT
GACTCACACCCCAAAGCACTCACCCACAACATCTGGAACCACGGGCACTATCACACATAGGTGTATATACAGACCCTTACACAGCA
ACAGCACTGGAACCTTCACAATTACATCCCCCCAAACCACACAGGCATAACTGATCATACGCAGCCTCAAGCAATGCCCAAAATAC
AAGTCAGACACAGCTTGTCAGAACACGCTCGTGTGCACGTACACACATGCAGCCCCTCCACTCTATCTCCTGAGTTCCATGAATACAC
ACCGACTCTCCAAGATGTACCCCACGTCTCACTTGCCACTGACCCCAGTTCCCTACCCACAAGCCCCAATCCATGCCTAAGCGTGGCC
CACAGAAGAACTTCTCTTTTATTTGGGATCCAAGGCCCCTGGCCCCCAGTGCCCATCCAATAAACTGTGGTCAGCTGGACAATCACCC
TGATCAGATATGGGAACATATAAGCAGACAGCTGGGTTTAAGATCCCAGCAGGAGAAAGCGGATACCAAATGAAAGAGAGTGCTAG
AACAGGTGCCTCAGCACTGTCTCCAGCACCCCAAATTCCTGCCTGTGGTTAGGAGACATCCATCAGGGCTCTAGGCCTCTCGGACCCG
GCCCAAGAGGCCAGCATTCTCCTGGCGAAGGGCTCGGTAGTCCTCACAGATCTTCTCCAGGTTGCTCAAAGTCTTCTTGCCCATCTCTG
TCTCAATCTAAGAAAACAGGATGCACACTTCTTCAGCCCCTGCAGGCTGCCCCTCTACTGAACTCCTCCCTGCTCCTCCTATTCCCGTA
ACAGCAGCCTGTTCCTTCCCATCACTGGGCTTCTGGGTATGTCCTTCCCTCCACTCCACCTAAAGCAGCAACTTCTGCCATGGGCTCTG
GGAGGCATTAGGAGCCGCAAGCTAAAAGCCAGGGCTCAGAGTAGGCTACTGGCTAGCTTCAGGTCCCAGGCACAGTGGGCACGAAG
GCAAAGCCTCTAGCTGTTAGTTGTCTGGTTTCAAAGACTCTCAGCGCAAAACAAGGAACTATCCCCTGGCCTGTCTCCATTCCCCTTAC
CAGTCCCAGGTCTCACCTGCTCCTCAAGATCTCGAACTTCCCTCATGATAGTGCCTGTGTCCTCAATGGTCTGGATGAGCTGACTGCAA
TTCTGGAGACAGCAAGAATACAAGGCTTGCACCTATGCTGGCCCTCTCCAGCCAACCCACCAGGCACATGGCTCCCCTCACCTCATG
CAGGGCAGCTAGGTACTTGTAGGCTTTCCGAACAGCATCATCCTTCTTAGCATCCTGATAAGACAAAGGGGATCTCCGAGATATCAGC
AAGCCATTCCCCCTTTTCCACTACTCTATGCCCCTATAAGACCACCCTTTACTAGTACTTTGCCTTCATCCTCCACAGAGCAAAGCTAG

GCCCCAAGCAACAGTGCACCTAAAGGACTCACAGAGGGGCAGGCAACAACTCAGTCCCGCCTCCACCCTCCCGGAGGCCAGCCTGC

TCCATACCTTGAACACAAGCTCATCAGTCACTGCAAATGTCCGGTCGAGCTTCCCAGAGAGAGAGTTGATTTCCTTCTGCAGTTCCTTT

GTGTCCGACAAGATCTGGTAGAAACCAGGGTAACTATCAGTGCACATCTTGGGCAAGGTAGCTGATCAGTGATAACACTCACGTGCC

TATACTTACATCCAGTCAGGGCCCATGTCGCTGTGTTGGGGTGACTATTATGTGTTGGAGTGTGCCTGAACAGCTCTGCCTAGTAGTGA

GCATAAAGTCCCTGTGTGATCACCCC

Figure S1. Primary sequence of the Foxp3 gene in FASTA format**.** Mus musculus strain C57BL / 6J chromosome X, GRCm38.p4 C57BL / 6J NCBI Reference Sequence: NC_000086.7. Exons (Red); Introns (black); In yellow, the complementary region of the anti-FoxP3 ASO (Intron 1).
